# On the Challenge of Fitting Tree Size Distributions in Ecology

**DOI:** 10.1371/journal.pone.0058036

**Published:** 2013-02-28

**Authors:** Franziska Taubert, Florian Hartig, Hans-Jürgen Dobner, Andreas Huth

**Affiliations:** 1 Department of Ecological Modelling, Helmholtz Centre for Environmental Research, Leipzig, Saxony, Germany; 2 Department of Biometry and Environmental System Analysis, Faculty of Forestry and Environmental Sciences, University of Freiburg, Freiburg, Baden-Wuerttemberg, Germany; 3 Faculty of Computer Science, Mathematics and Natural Sciences, University of Applied Science, Leipzig, Saxony, Germany; University of California, Irvine, United States of America

## Abstract

Patterns that resemble strongly skewed size distributions are frequently observed in ecology. A typical example represents tree size distributions of stem diameters. Empirical tests of ecological theories predicting their parameters have been conducted, but the results are difficult to interpret because the statistical methods that are applied to fit such decaying size distributions vary. In addition, binning of field data as well as measurement errors might potentially bias parameter estimates. Here, we compare three different methods for parameter estimation – the common maximum likelihood estimation (MLE) and two modified types of MLE correcting for binning of observations or random measurement errors. We test whether three typical frequency distributions, namely the power-law, negative exponential and Weibull distribution can be precisely identified, and how parameter estimates are biased when observations are additionally either binned or contain measurement error. We show that uncorrected MLE already loses the ability to discern functional form and parameters at relatively small levels of uncertainties. The modified MLE methods that consider such uncertainties (either binning or measurement error) are comparatively much more robust. We conclude that it is important to reduce binning of observations, if possible, and to quantify observation accuracy in empirical studies for fitting strongly skewed size distributions. In general, modified MLE methods that correct binning or measurement errors can be applied to ensure reliable results.

## Introduction

Strongly skewed size distributions occur in a wide range of natural systems. Examples include search patterns in animals known as Lévy flights [1]–[5], frequency distribution of earthquake magnitudes [6] and fire sizes [7], [8], and the relation of species abundances to their individual body size [9]–[11], in particular, stem size distributions of trees [12]–[16]. Several studies, for example the self-organized criticality (e.g. applied to forest fires), or metabolic theories, focus on the nature of the processes that underlie such size distributions and make specific predictions about the functional form and associated parameters [10], [11], [17]–[19]. For example, Enquist & Niklas [14] propose a power-law distribution with a scaling parameter 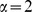 for the stem size frequency distribution of natural forests [10].

**Figure 1:**
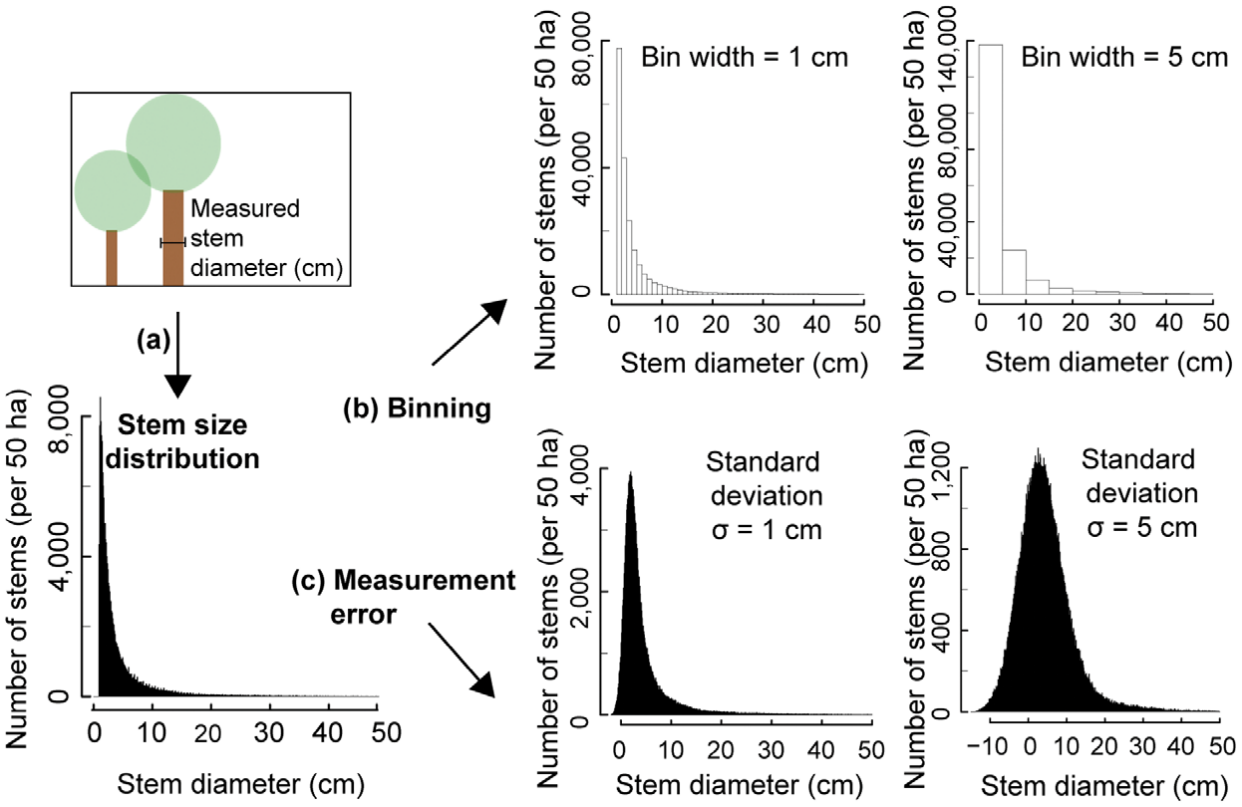
Outline of tree size measurements in forests. (a) In general, the stem diameter of a tree is measured at breast height (1.3 m). Each tree in the area of interest is tagged, recorded and measured. Using a specific class width (here 1 mm) each measured stem diameter is classified in its corresponding class. This results in a number of stems per class and is summarized in a stem size distribution. (b)-(c) Change of the functional form of the stem size distribution of stem diameters under binning or including measurement errors. (b) Change of the stem size distribution using binning of measured stem diameters with bin widths of 1 cm and 5 cm. (c) Change of the stem size distribution adding random measurement errors of standard deviations 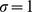 cm and 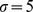 cm to the recorded stem diameters.

When testing theoretical predictions, we have to consider that field data contain uncertainties. For example, in forest science field data on tree size are typically analysed by constructing a stem size frequency distribution which summarizes the number of trees in different measured stem diameter classes (Fig. 1a). Such a classification of the measured data into diameter classes of a certain width is also called *binning* of data. Thus, results of analyses depend on the class width, whereby in forestry widths of 5 cm or 10 cm are often used. Besides the influence of binning, uncertainties in field data can also arise from irregularities or errors that occur during the measurement process [20]. Such *measurement errors* typically lead to a symmetric variation around the true value. Both binning and measurement errors change the functional shape of the analysed frequency distribution (Fig. 1b, 1c).

Two methods are mainly used to estimate the parameters of size distributions - maximum likelihood estimation (MLE) and linear regression. Linear regression can only be applied to pre-binned data and thus, leads to serious complications not only in assessing parameters [2], [21], but also in determining the correct corresponding distribution as the best fit using the coefficient of determination r^2^ (Franziska Taubert, unpublished data). Instead, MLE is known to be the most accurate approach to date as it does not require pre-binned data and thus, shows numerous advantages, for example, low bias and low variance of parameter estimates [2], [21], [22]. Nevertheless, linear regression is still used [3], [10]. However, even when MLE is applied, difficulties may also arise when there are observation uncertainties in the data.

In this study we analyse how parameter estimation and the selection of the true corresponding frequency distribution are affected by (a) binning and (b) random measurement errors. As far as we know, no previous study has systematically examined the effect of binning and random measurement errors on MLE parameter estimates and distribution selection results for decaying size distributions in ecology. To account for binning and to correct random measurement errors, we propose modified MLE methods. Using large virtual data sets produced from three distributions (power-law, negative exponential and Weibull distribution) we also test whether potential effects can be corrected by these modified methods. We investigate the following questions:

1. Which effects do observation uncertainties have on parameter estimates and on determining the underlying frequency distribution when uncertainties are not considered in the MLE method?
2. To what extent do the two modified MLE methods reduce potential effects in parameter estimation?
3. Which advantages do the two modified MLE methods show in determining the frequency distribution that underlies the observations?

Finally, we demonstrate the application of the investigated methods on a large field data set of measured stem diameters for a tropical forest.

## Materials and Methods

### Maximum Likelihood Estimation

In this study, we use maximum likelihood estimation (MLE) for inferring parameters of frequency distributions. Given a sample 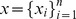 of observations, the likelihood *L* is defined as the probability of obtaining these measured field data. Assuming that the data points are independent, *L* can also be written as the product of the single probabilities 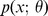 of each data point depending on unknown parameters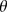:
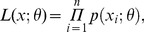
(1)where 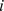 is indexing the corresponding observation points. To estimate the unknown parameters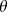, the likelihood 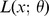 is maximized.

Different types of assumptions can be made for the probability 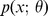 of a measured data point. Most simple is the presumption that this probability is given by an assumed frequency distribution 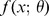 without observation uncertainties. Therefore, 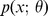 is simply replaced by the assumed frequency distribution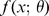.
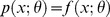
(2)


In the following, we call this method *standard MLE*.

Generally, *standard MLE* is applied to continuous data. But, field data often show inaccuracies. Such data inaccuracies occur either as binning (e.g. rounding measured data) or as random measurement errors (e.g. non-systematic uncertainties). Binning 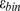 equals a classification of data into half-open intervals of width 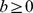 cm. Measurement stochasticity 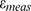 is typically assumed to be Gaussian distributed with mean 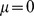 cm and standard deviation 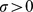 cm.

To account for binning of data, the multinomial approach is used to describe the expected probability of observing a single data point within a class of a certain width 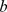 (cm).This probability depends on the assumed frequency distribution 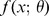 (Methods S1).
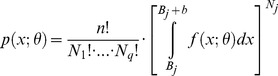
(3)with the 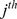 bin denoted as 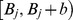 and 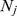 as the number of observations falling in the corresponding bin. Altogether there are 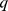 classes, where 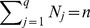 is the total number of observations. A few studies have already followed this approach [1], [15]. Here, we call this MLE which considers binning uncertainties the *multinomial MLE*.

For correcting measurement errors we use a hierarchical fitting function: first it is assumed that the data points originate from the presumed frequency distribution 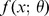 and are then perturbed by a random measurement error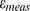.
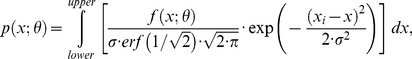
(4)where 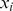 stands for the 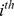 observation value, 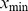 and 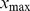 correspond to their minimum and maximum and 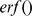 refers to the Gauss error function. In detail, we assume for the measurement error a truncated Gaussian distribution with mean 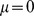 cm and constant standard deviation 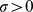 cm. We set the truncation at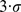, which results in limits of the integral (eqn 4) of 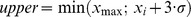 and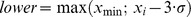. As before, for the purpose of this paper we refer to this MLE, which amends measurement errors, as the *Gaussian MLE*.

### Virtual Data Sets

A power-law distribution is mostly used to fit strongly skewed frequency distributions [1], [3], [23]. However, a typical question is whether a given empirical distribution is really best described by a power-law distribution, or whether similar frequency distributions such as a negative exponential distribution also provide a good fit. Therefore we concentrate here not only on the power-law, but also on the negative exponential distribution and the Weibull distribution. We include the Weibull distribution because some studies take it into account to possibly describe a size distribution, for example, of tree diameters [15], [24], [25]. In general, our results will qualitatively apply to most functions that depict strongly skewed distributions.

To test the MLE methods, we generate 1,000 virtual data sets of sample size 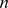 from each assumed frequency distribution 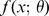 using the *inverse transformation method* (Methods S1). Parameters of these distributions are set as follows:

- scaling parameter 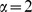 for the power-law distribution,
- parameter 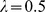 for the negative exponential distribution and
- parameters 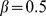 and 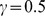 for the Weibull distribution.

We choose an exponent of 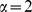 for the power-law distribution because this value is suggested by Enquist & Niklas [14] for the stem size frequency distribution of natural forests. Parameters of the other distributions are chosen in a way that the shape of the probability density function is comparable to those of the power-law distribution. We assume that these three distributions are truncated in the range of 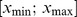 (Table 1). We set 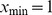 cm and 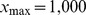 cm throughout the evaluations (typical values for tree size distributions).

**Table 1:** Presentation of the three assumed truncated frequency distributions 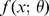 used in our investigations.

frequency distribution	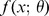
power-law distribution	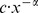
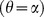	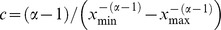 with
exponential distribution	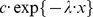
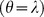	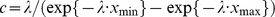 with
Weibull distribution	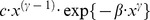
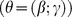	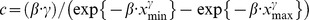 with

To assess the accuracy of MLE for imprecise data, we either apply binning to the virtual samples or overlay them with a measurement error. Concerning binning, we increase the width 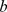 from 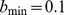 cm to 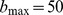 cm with a step size of 0.1 cm. For measurement errors we randomly generate values from a Gaussian distribution with 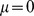 cm and 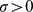 cm and add them to the produced virtual data. The parameter 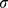 of 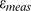 we use in our investigations ranges from 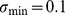 cm to 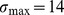 cm increasing with a step size of 0.1 cm. For the example of stem diameter distributions in forestry, a standard deviation 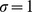 cm results in an expected average deviation of 20% for stem diameters of 5 cm. Finally, we evaluate each sample applying the three MLE methods (eqn 2 to eqn 4). We also vary the sample size 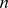 of the produced virtual data (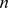 = 100; 500; 1,000; 5,000; 10,000; 50,000) to check for an effect of sample size on estimation. Due to computational limitations, we reduce repetitions and sample size for the *Gaussian MLE*, for which we only analyse 250 samples (of sample size 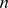 = 100; 500).

**Figure 2:**
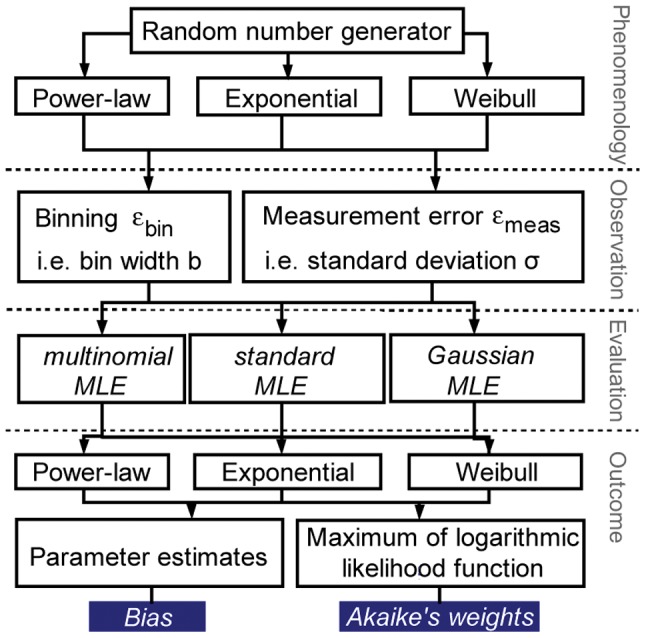
Scheme of the evaluation procedure of virtual data sets.

The calculations result in parameter values for each distribution dependent on 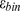 or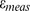. We fit the raw and modified virtual data sets by applying *standard MLE* as well as *multinomial MLE* or *Gaussian MLE*. This allows us to compare the estimation bias for each type of observation uncertainty and offers the opportunity to evaluate the capability of error correction (Fig. 2). For the binned virtual data we use the centre of the bins as data values when evaluated with the *standard MLE*.

To evaluate which of the supposed distributions 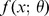 best represents a specific data sample, we choose *Akaike weights* [26]. The distribution with the highest *Akaike weight* expresses the data best according to the set of the supposed distributions.

To apply our methods to real-world data, we use data from a forest inventory on Barro Colorado Island (BCI) from the year 2000 [27]–[29]. Stem diameter measurements are recorded as integers (in mm) at breast height (1.3 m). Here, we report data values in (cm). We only take into account those measured trees that are declared as “alive” and as “main stems”. We exclude measurements of the smallest possible recorded diameter value (1 cm) to avoid distortions due to uncertainty about rounding for the smallest values [15]. Minimum and maximum measurements are set to 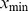 and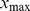, encompassing in total 207,105 observations. Bin width is documented as 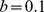 cm. The measurement error has been estimated by repeated measurements of 1,715 trees [20], [27]. The corresponding deviations have been fitted with a sum of two Gaussian distributions. The first Gaussian distribution depicts small deviations increasing with stem diameter in (cm) (mean 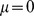 cm; standard deviation 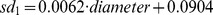 cm), according to 95% of the observed trees. The second Gaussian distribution describes larger ones (mean 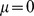 cm; standard deviation 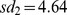 cm), associated with the remaining 5% of trees [20].

All evaluations of the virtual and BCI data are performed with R-2.10.0 [30]. For MLE optimization of the power-law or exponential distribution, we employ a combination of golden section search and successive parabolic interpolation [31], [32]; for the Weibull distribution, we choose the *Nelder-Mead algorithm* [33], [34]. In cases of convergence difficulties for Weibull distributed data, we change the optimization technique to the *L-BFGS-B algorithm* [34], [35]. All optimization algorithms used are already implemented in R-2.10.0.

## Results

### Effect of Binning and Measurement Errors

**Figure 3:**
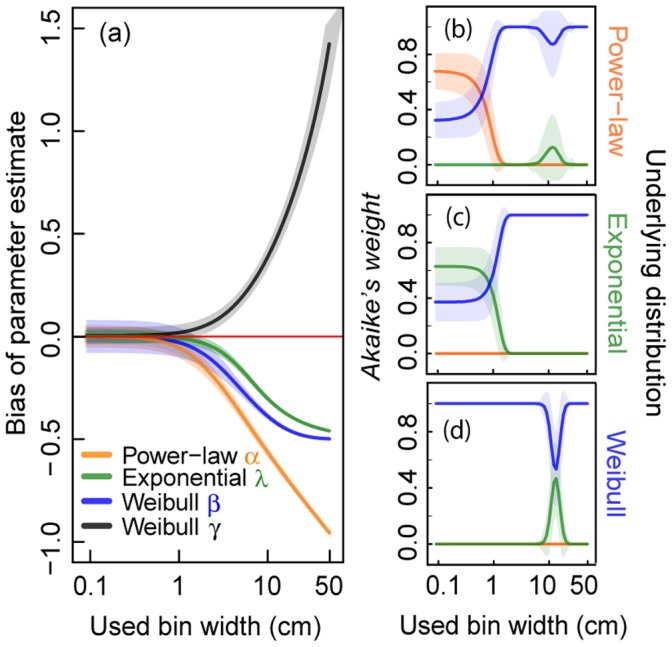
Analyses of binned virtual data using different bin widths. We evaluate 1,000 virtual data sets of sample size 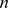 = 500 from a truncated power-law, a truncated negative exponential and a truncated Weibull distribution. Virtual data are classified into classes of certain bin width (x-axis in cm) before applying *standard MLE*. (a) Effect of binning on parameter estimates of the three investigated distributions. (b)–(d) Effect of binning on *Akaike weights* supposing three distributions (power-law, negative exponential and Weibull distribution) for (b) power-law distributed virtual data, (c) negative exponentially distributed virtual data and (d) Weibull distributed virtual data. The highest *Akaike weight* determines the best fit of a frequency distribution to the data. Solid lines represent the mean values and shaded areas show the standard deviation (of 1,000 calculated values).

Increasing bin widths generally affects the parameter estimates of all three considered distributions, thus creating remarkable biases (Fig. 3a). Based on representative virtual data of sample size 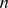 = 500, only small bin widths of approximately 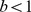 cm ensure a mean bias of less than 5% of the true parameter of the corresponding distributions (Table S1). With incrementing widths of 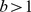 cm, nearly all parameters are on average underestimated, except the parameter 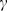 of the Weibull distribution, which is highly overestimated (Fig. 3a). Maximum absolute values of the mean bias range from 48% (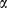-estimates) to 280% (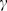-estimates) (Table S1). Standard deviations of 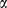-, 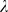
- and 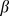-parameter estimates decrease with bin width, whereas the standard deviation of 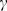-values increases (Fig. 3a).

**Figure 4:**
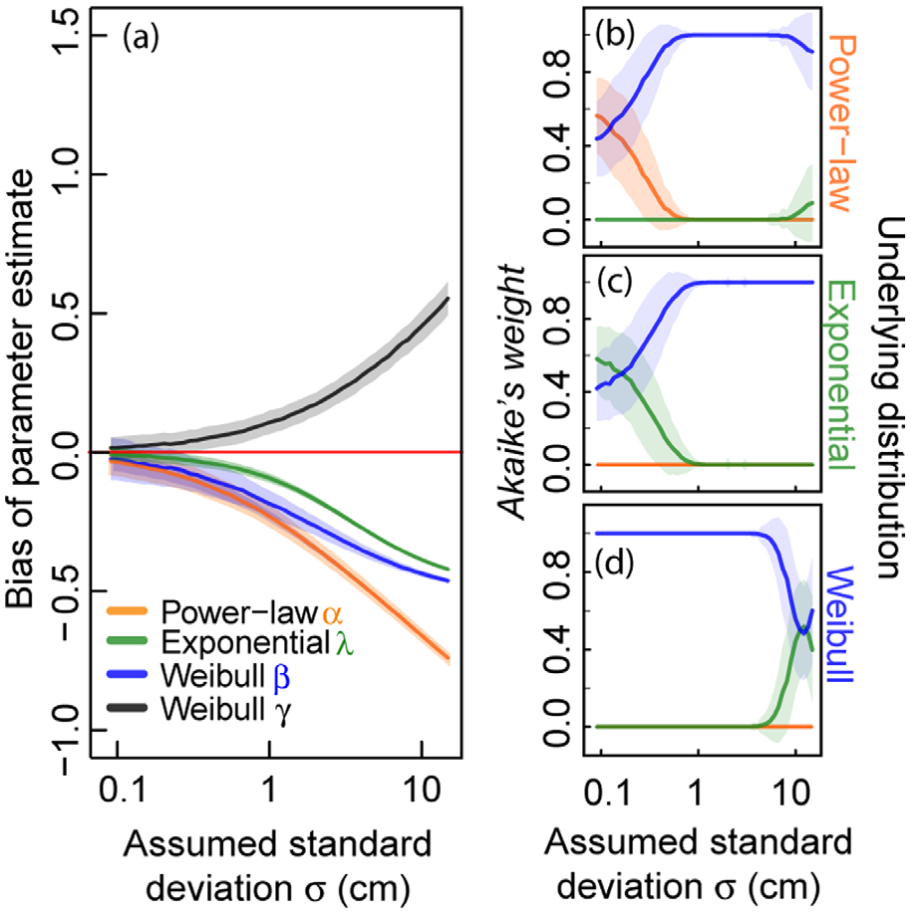
Analyses of virtual data including different levels of measurement errors. We evaluate 1,000 virtual data sets of sample size 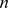 = 500 from a truncated power-law, a truncated negative exponential and a truncated Weibull distribution. An error value generated from a Gaussian distribution with mean 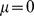 cm and an assumed standard deviation 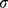 (x-axis in cm) is added to each virtual data point before applying *standard MLE*. (a) Effect of random measurement errors on parameter estimates of the three investigated distributions. (b)–(d) Effect of random measurement errors on *Akaike weights* supposing three distributions (power-law, negative exponential and Weibull distribution) for (b) power-law distributed virtual data, (c) negative exponentially distributed virtual data and (d) Weibull distributed virtual data. The highest *Akaike weight* determines the best fit of a frequency distribution to the data. Solid lines represent the mean values and shaded areas show the standard deviation (of 1,000 calculated values).

Random measurement errors included in the virtual data sets with 500 values also have substantial effects on parameter estimates (Fig. 4a). For 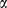-, 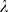
- and 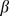-estimates the mean parameter value is underestimated (again, except for the parameter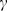). Significant effects already start at a small measurement error of 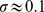 cm with a mean bias of approximately 5% of the true parameter value (Fig. 4a, Table S1). Absolute mean biases reach their maximum in the range between 37% (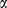-estimates) and 110% (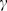-estimates) (Table S1). Standard deviations of parameter estimates show similar trends as was observed for binned data.

Binning strongly affects the correct determination of a power-law distribution. Only for small bin widths (<0.67 cm) can the correct distribution be identified using *Akaike weights* (Fig. 3b, Table S1). Thereby, the distribution with the highest weight best represents the data with regard to the set of the three supposed distributions. For widths above this threshold, an increasing chance of selecting a Weibull distribution occurs instead (Fig. 3b). Surprisingly, this effect is not improved by increasing the sample size (Fig. S1). Looking at exponentially distributed data, the true distribution cannot be distinguished from the Weibull distribution with high certainty even when the data are not binned. For bin widths below approximately 0.91 cm the probability of correct identification is on average higher than 50% (Fig. 3c, Table S1). Above this threshold, the probability of selecting a Weibull distribution instead increases strongly. Again, this problem is not solved by increasing the sample size (Fig. S1). Binning of Weibull distributed data does not influence the determination of the correct distribution over a large range of bin widths (Fig. 3d, Table S1). But, for bin widths between approximately 11 cm and 15 cm there is a small chance of wrongly selecting an exponential distribution. With increasing sample size, this small probability of false selection decreases (Fig. S1). Note that the Weibull distribution is more flexible than the other two as it includes one additional parameter.

If we include measurement errors in the raw data, the determination of the correct distribution using *Akaike weights* based on the *standard MLE* method shows different results than for binning (Fig. 4b, 4c, 4d). Only for small measurement errors of 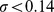 cm can a power-law be identified correctly by looking at the mean *Akaike weights* (Fig. 4b, Table S1). For assumed standard deviations 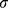 greater than this threshold, a steeply increasing probability of determining a Weibull distribution is observed. An exponential distribution can only be detected for a small measurement error of 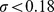 cm (Fig. 4c, Table S1). Weibull distributions are in most cases correctly identified, except for very large measurement errors (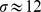 cm) (Fig. 4d, Table S1). At this value, the chance of selecting an exponential distribution increases. Similar effects can be observed for the data sets with higher sample size (Fig. S3).

### Performance of Modified MLE Methods

**Figure 5:**
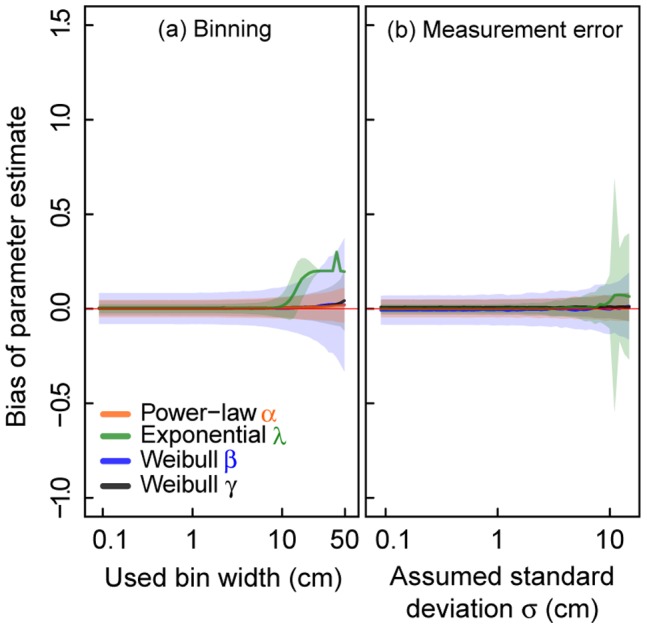
Effects of binning and random measurement errors on parameter estimation using different MLE methods. (a) MLE including binning (*multinomial MLE*) and (b) MLE accounting for measurement errors (*Gaussian MLE*). We evaluate virtual data sets of sample size 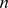 = 500 from a truncated power-law, a truncated negative exponential and a truncated Weibull distribution. Solid lines represent the mean estimates and shaded areas show the standard deviation (of (a) 1000 values and (b) 250 values). (a) Effect of binning on parameter estimates. Virtual data are classified into classes of certain bin width (x-axis in cm). (b) Effect of random measurement errors on parameter estimates. An error value generated from a Gaussian distribution with mean 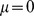 cm and an assumed standard deviation 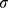 (x-axis in cm) is added to each virtual data value.

Using *multinomial MLE*, the negative effects can be reduced to a large extent (Fig. 5a, Table S1). For the entire range of investigated bin widths, a significantly lower mean bias of 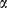-, 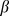
- and 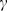-parameter estimates can be observed not exceeding a mean bias of 9% of the corresponding true parameter value (Table S1). For 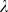-estimates binning correction fails only for high widths (>11 cm, Fig. 5a). However, it reaches a maximum absolute mean bias of 59% of the true 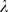-value, which is still smaller than for employing *standard MLE* (Table S1). Standard deviations of the parameter estimates increase with increasing bin width for nearly all parameters, except for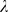, which decreases (Fig. 5a).

For data overlaid with a measurement error, the *Gaussian MLE* provides significantly better results than the *standard MLE* (Fig. 5b). The mean bias remains below 3% of the true 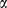-, 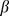
- and 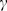-parameter (Table S1). For a large range of measurement errors (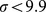 cm), 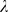-estimates are within the 5% mean bias threshold. But for increasing errors of 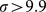 cm, also the *Gaussian MLE* produces a higher mean bias, reaching up to 14% of the true 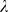-parameter value (Table S1).

### Determination of the Correct Frequency Distribution

**Figure 6:**
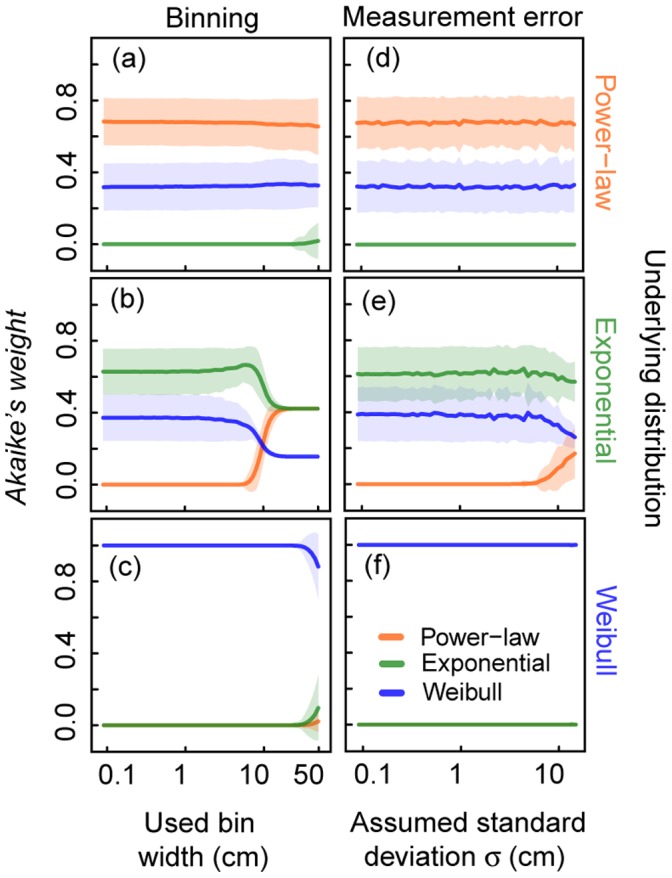
Effect of errors on *Akaike weights* for the correct determination of the underlying distribution. In each row virtual data sets of sample size 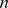 = 500 which originate from the three truncated distributions (power-law, negative exponential and Weibull distribution) are evaluated. Weights are calculated supposing these distributions (power-law, negative exponential and Weibull distribution) with (a)–(c) *multinomial MLE* and (d)–(f) *Gaussian MLE*. The highest *Akaike weight* determines the best fit of a frequency distribution to the data. (a)–(c) Effect of binning of virtual data sets with used bin width (x-axis in cm) on *Akaike weights*. (d)–(f) Effect of random measurement errors added to the virtual data sets on *Akaike weights*, whereby errors are Gaussian distributed with mean 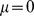 cm and assumed standard deviation 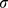 (x-axis in cm). Solid lines represent the mean of *Akaike weights* and shaded areas show the standard deviation (of (a)–(c) 1000 values and (d)–(f) 250 values).

The identification of the underlying distribution with MLE including observation uncertainties (*multinomial MLE* and *Gaussian MLE*) shows a significant improvement compared to *standard MLE* (Fig. 6). An underlying power-law or Weibull distribution is always correctly determined (Fig. 6a, 6c, 6d, 6f). For exponentially distributed data, the correct distribution is identified with at least 50% probability for a large range of bin widths (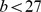 cm, Table S1). Above this threshold, *Akaike weights* favour a power-law distribution (Fig. 6b). Concerning measurement errors, the exponential distribution is identified for all measurement errors (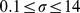) in the range of our investigations (Fig. 6e). An increment in sample size has considerable positive effects for both modified MLE methods (Fig. S2, S4).

### Application: Stem Size Distribution of a Tropical Forest

We now employ the investigated fitting methods on forest inventory data, here on measured stem diameters of a tropical rainforest (207,105 observations).

We apply *standard*, *multinomial* and *Gaussian MLE* to the field data supposing a truncated power-law, a negative exponential and a Weibull distribution (Table 1; Code S1). For comparison, we also estimated (using algorithms implemented in R-2.10.0) the parameters of (a) a truncated power-law distribution with linear regression on log-log axes, (b) a truncated negative exponential distribution with linear regression on a logarithmic y-axis of stem frequencies, and (c) a truncated Weibull distribution with nonlinear regression on log-log axes.

MLE parameter estimates do not differ significantly according to the different methods used (whether observation uncertainty was accounted for or not). For each supposed distribution according to the different methods (in brackets from left to right: *standard MLE*, *multinomial MLE*, *Gaussian MLE*) estimates are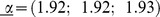, 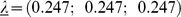, 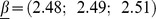 and 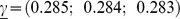. These results fit well to our findings where we showed that for a width of 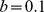 cm no significant difference in the mean estimates using *standard* or *multinomial MLE* is expected. Additionally, we showed that for small measurement errors of 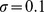 cm only small biases are expected using *standard MLE* compared to *Gaussian MLE*. The stem diameter of 80% of the BCI data is less than or equal to 5.8 cm and thus, a small estimated measurement error of less than 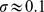 (with 95% probability) is expected.

Results of regression methods differ significantly from those of the MLE methods (Fig. S5). Regression provides the following estimates of parameters compared to *standard MLE* (in brackets from left to right: regression, *standard MLE*):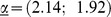, 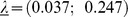, 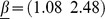 and 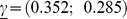. Additionally, linear regression favours the truncated power-law distribution and *standard MLE* the truncated Weibull distribution. The residual standard error or determination coefficient r^2^ used within regression does not always reliably determine the underlying distribution (Franziska Taubert, unpublished data).

## Discussion

Maximum likelihood estimation (MLE) has been recommended for fitting size distributions by several authors [2], [21], [22]. In this study, we investigated the effects of different types of uncertainties on the estimation procedure using MLE. We focused on the bias of parameter estimates and on the reliability to determine the underlying frequency distribution using *Akaike weights*. Our results show that using MLE without correcting uncertainties does not solve the main problems arising when estimating parameters of strongly skewed size distributions. This method is appropriate as long as uncertainties in the observations do not have a great influence. However, even when the underlying ecological process can be described well by a strongly skewed frequency distribution, random errors and rounding in the data acquisition process can lead to biased parameter estimates and falsely selected distributions. In these cases, we recommend the use of modified MLE methods for including observation uncertainty.

A problem that arises in practical applications that we have not addressed in this study is the estimation of the truncation parameters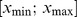. In particular, it is known that the definition of 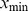 influences the fitting results [22]. Also the upper truncation parameter 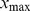 has an effect on the fitting. One could estimate both parameters in such a way that the interval 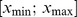 covers only a section of the entire empirical size distribution. Fitting only such a section would lead to high biases in the estimation of parameters and in the selection of the best fitting frequency distribution. Fitting segments of size distributions caused either by estimating a narrow interval 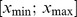 or by assuming a composite function to describe the size distribution, are not further discussed here. Related investigations concerning binning can be found elsewhere [36].

In our investigations we used *Akaike weights*, based on the *Akaike Information Criteria (AIC)*, to select the best fitting frequency distribution from our three assumed skewed, decaying distributions. The *AIC* may cause some difficulties, for example, when data values are not independent of each other [37]. Additionally, the *AIC* does not consider sample size in its calculation. Nevertheless, the *AIC* is an often used criterion for model selection in ecological studies. Please notice also, the *AIC* is only a criterion for model selection, but does not ensure that the best fitting frequency distribution is in fact the true underlying one. For this purpose, hypothesis tests are recommended.

Regarding the types of uncertainties and their strength (i.e. bin width 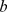 and measurement error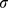) we assume them to be known in our study. However, in practice this may often not be the case. Random measurement errors can be detected in the field by repeated measurements [20], [27]. However, errors may also be hidden in such repeated measurements, similar or different to those in the first observations. For example, similar errors might occur due to irregularities in the observation object. In general, it cannot be guaranteed that all possible sources of random errors are captured correctly or that each can be assumed to be Gaussian distributed.

In practice, both systematic and stochastic observation uncertainties will often appear together, also with differing relative importance. For example, field measurements of tree diameter with high measurement precision may be more affected by stochastic measurement errors. On the other hand, if field observations are measured using pre-defined bins with a width of, for example, 5 cm or 10 cm, the effects of binning are expected to be greater than those of random measurement errors. If equally great effects of these two observation uncertainties are present, it might be necessary to consider both. Therefore, another modified MLE method should be created to include both uncertainties. Further investigations are needed to determine whether such an MLE method would show an advantage over those MLEs that correct only one type of observation uncertainty.

Nevertheless, these limitations do not alter our general findings, namely, that uncertainties in the observation process lead to serious difficulties in the correct determination of the underlying frequency distribution and in the estimation of its parameters. This makes comparing inferred parameters across data sets or with ecological theory difficult. Modified MLE methods that are discussed in this paper lead to significantly better parameter estimates and more reliable identifications of frequency distributions underlying size distributions.

## Supporting Information

Figure S1 **Effect of binning on** ***Akaike weights*** **with increasing sample size using** ***standard MLE.*** Weights are calculated with MLE assuming perfect observations (*standard MLE*) dependent on the used bin width *b* (x-axis in cm). The highest *Akaike weight* determines the best fit of a frequency distribution to the data. The evaluated virtual data sets originate from the three truncated distributions (per column from left to right: power-law, negative exponential and Weibull distribution) which underlie them. Rows from top to bottom: Effect of binning on the identification of the correct distribution based on virtual data of sample size *n* = 100; 500; 1,000; 5,000; 10,000 and 50,000. Solid lines represent the mean *Akaike weights* and shaded areas show the standard deviation (of 1,000 calculated values).
(TIF)

Figure S2 **Effect of binning on** ***Akaike weights*** **with increasing sample size using** ***multinomial MLE*** **.** Weights are calculated with MLE accounting for binning (*multinomial MLE*) dependent on the used bin width *b* (x-axis). The highest *Akaike weight* determines the best fit of a frequency distribution to the data. The evaluated virtual data sets originate from the three truncated distributions (per column from left to right: power-law, negative exponential and Weibull distribution) which underlie them. Rows from top to bottom: Effect of binning on the identification of the correct distribution based on virtual data of sample size *n* = 100; 500; 1,000; 5,000; 10,000 and 50,000. Solid lines represent the mean *Akaike weights* and shaded areas show the standard deviation (of 1,000 calculated values).
(TIF)

Figure S3
**Effect of random measurement errors on** ***Akaike weights*** **with increasing sample size using** ***standard MLE*** **.** Weights are calculated with MLE assuming perfect observations (*standard MLE*) dependent on the Gaussian distributed errors with mean 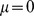 cm and assumed standard deviation 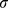 (x-axis in cm). The highest *Akaike weight* determines the best fit of a frequency distribution to the data. The evaluated virtual data sets originate from the three truncated distributions (per column from left to right: power-law, negative exponential and Weibull distribution) which underlie them. Rows from top to bottom: Effect of measurement errors on the identification of the correct distribution based on virtual data of sample size *n* = 100; 500; 1,000; 5,000; 10,000 and 50,000. Solid lines represent the mean *Akaike weights* and shaded areas show the standard deviation (of 1,000 calculated values).(TIF)

Figure S4
**Effect of random measurement errors on** ***Akaike weights*** **with increasing sample size using** ***Gaussian MLE*** **.** Weights are calculated with MLE assuming measurement errors (*Gaussian MLE*) dependent on the Gaussian distributed errors with mean 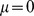 cm and assumed standard deviation 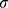 (x-axis in cm). The highest *Akaike weight* determines the best fit of a frequency distribution to the data. The evaluated virtual data sets originate from the three truncated distributions (per column from left to right: power-law, negative exponential and Weibull distribution) which underlie them. Top: Effect of measurement errors on the identification of the correct distribution based on virtual data of sample size *n* = 100. Bottom: Effect of measurement errors on the identification of the correct distribution based on virtual data of sample size *n* = 500. Solid lines represent the mean *Akaike weights* and shaded areas show the standard deviation (of 250 calculated values).(TIF)

Figure S5
**Log-log plots of the fits using regression (right) and** ***Gaussian MLE*** **(left).** Data values (inventory data from Barro Colorado Island) of measured stem diameter (cm) at breast height (1.3 m) are shown as black points and fitted truncated distribution functions are represented by solid lines. The straight line denotes the power-law (orange), the slightly curved line refers to the Weibull distribution (blue) and the stronger curved line depicts the negative exponential distribution function (green). Estimated parameters are for (right) regression 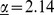 (power-law), 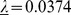 (exponential distribution), 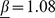 and 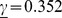 (Weibull distribution) and for (left) *Gaussian MLE* 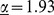 (power-law), 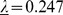 (exponential distribution), 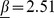 and 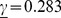 (Weibull distribution).(TIF)

Table S1
**Specific key points of the evaluation of the virtual data samples.** (i) bin width *b* or standard deviation 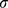 at which the mean bias is greater than or equal to 5% of the true parameter value, (ii) maximum absolute value of the mean bias as percentage of the true parameter value and (iii) the bin width 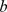 or standard deviation 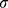 at which the next best distribution reveals the same or a higher mean *Akaike weight*. We consider the three truncated frequency distributions (power-law, negative exponential and Weibull distribution) and evaluate virtual data samples produced from these distributions using *standard MLE* (assuming no observation uncertainties), *multinomial MLE* (correcting binning of data) and *Gaussian MLE* (correcting measurement errors).(DOC)

Methods S1 **Details on the evaluation procedure and formulas.**
(DOC)

Code S1 **R-script of MLE evaluation on the example of Barro Colorado Island census year 2000.**
(DOC)
