# Supplementary figures and images for: On the Challenge of Fitting Tree Size Distributions in Ecology
Source: PLoS One. 2013 Feb 28;8(2):e58036. doi: 10.1371/journal.pone.0058036 (PMC3585190; doi:10.1371/journal.pone.0058036)

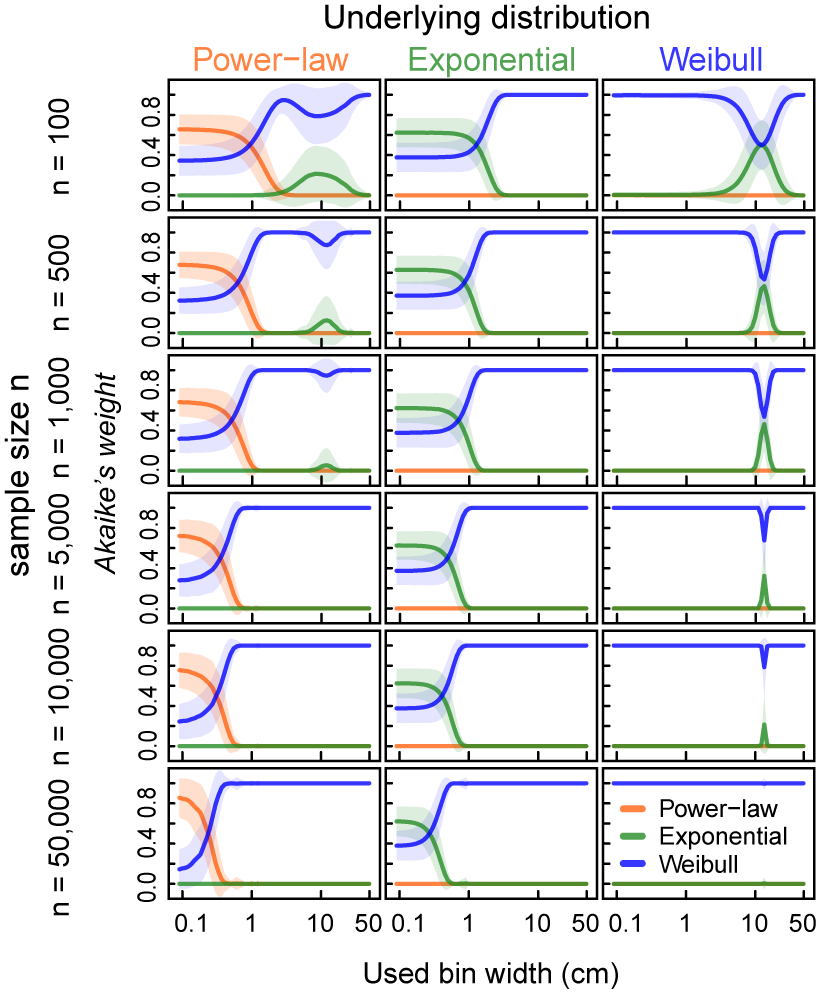

Supplement: Figure S1 — Effect of binning on Akaike weights with increasing sample size using standard MLE. Weights are calculated with MLE assuming perfect observations (standard MLE) dependent on the used bin width b (x-axis in cm). The highest Akaike weight determines the best fit of a frequency distribution to the data. The evaluated virtual data sets originate from the three truncated distributions (per column from left to right: power-law, negative exponential and Weibull distribution) which underlie them. Rows from top to bottom: Effect of binning on the identification of the correct distribution based on virtual data of sample size n = 100; 500; 1,000; 5,000; 10,000 and 50,000. Solid lines represent the mean Akaike weights and shaded areas show the standard deviation (of 1,000 calculated values). (TIF) [file pone.0058036.s001.tif]

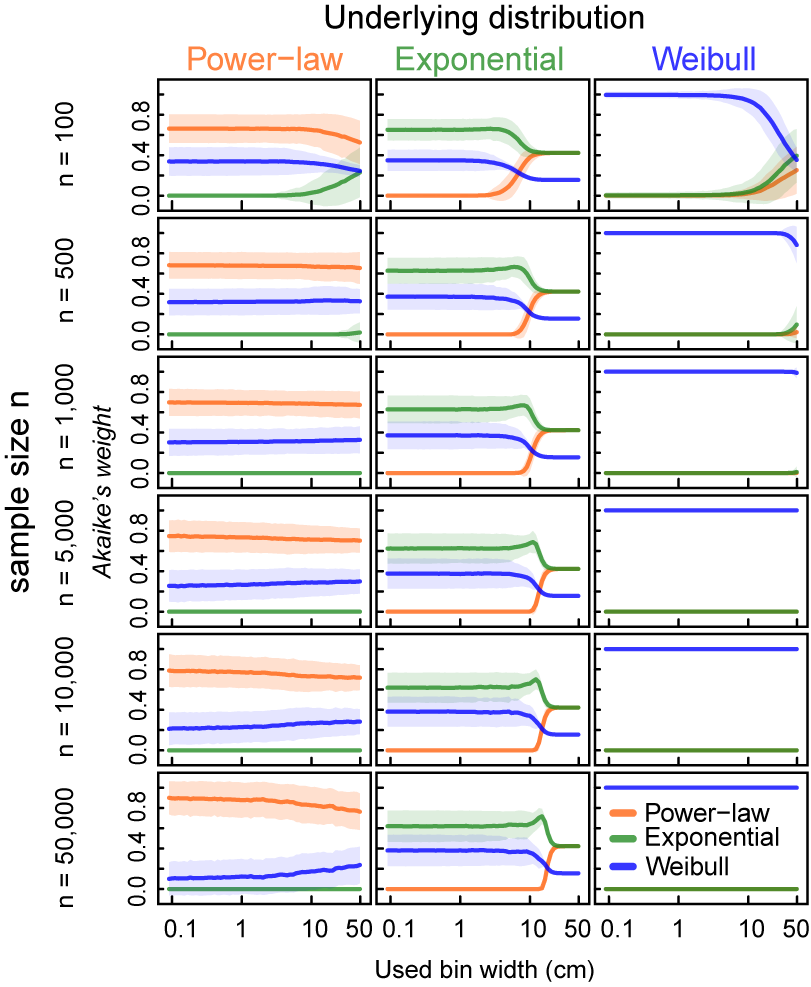

Supplement: Figure S2 — Effect of binning on Akaike weights with increasing sample size using multinomial MLE . Weights are calculated with MLE accounting for binning (multinomial MLE) dependent on the used bin width b (x-axis). The highest Akaike weight determines the best fit of a frequency distribution to the data. The evaluated virtual data sets originate from the three truncated distributions (per column from left to right: power-law, negative exponential and Weibull distribution) which underlie them. Rows from top to bottom: Effect of binning on the identification of the correct distribution based on virtual data of sample size n = 100; 500; 1,000; 5,000; 10,000 and 50,000. Solid lines represent the mean Akaike weights and shaded areas show the standard deviation (of 1,000 calculated values). (TIF) [file pone.0058036.s002.tif]

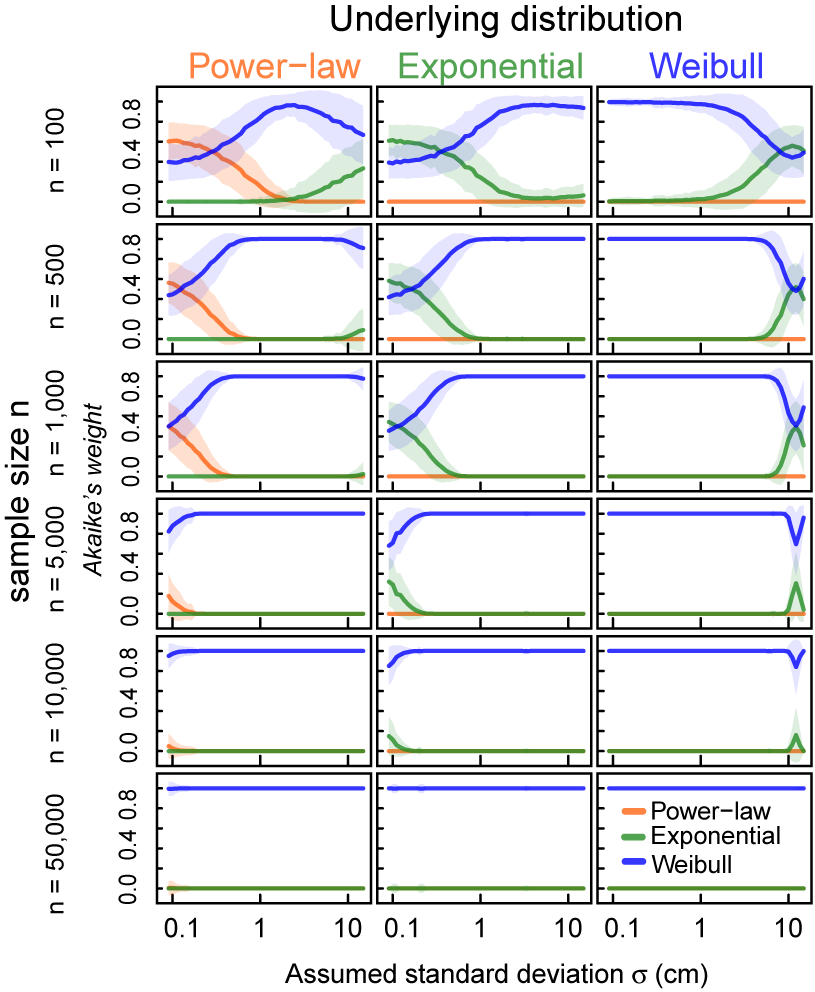

Supplement: Figure S3 — Effect of random measurement errors on Akaike weights with increasing sample size using standard MLE . Weights are calculated with MLE assuming perfect observations (standard MLE) dependent on the Gaussian distributed errors with mean cm and assumed standard deviation (x-axis in cm). The highest Akaike weight determines the best fit of a frequency distribution to the data. The evaluated virtual data sets originate from the three truncated distributions (per column from left to right: power-law, negative exponential and Weibull distribution) which underlie them. Rows from top to bottom: Effect of measurement errors on the identification of the correct distribution based on virtual data of sample size n = 100; 500; 1,000; 5,000; 10,000 and 50,000. Solid lines represent the mean Akaike weights and shaded areas show the standard deviation (of 1,000 calculated values). (TIF) [file pone.0058036.s003.tif]

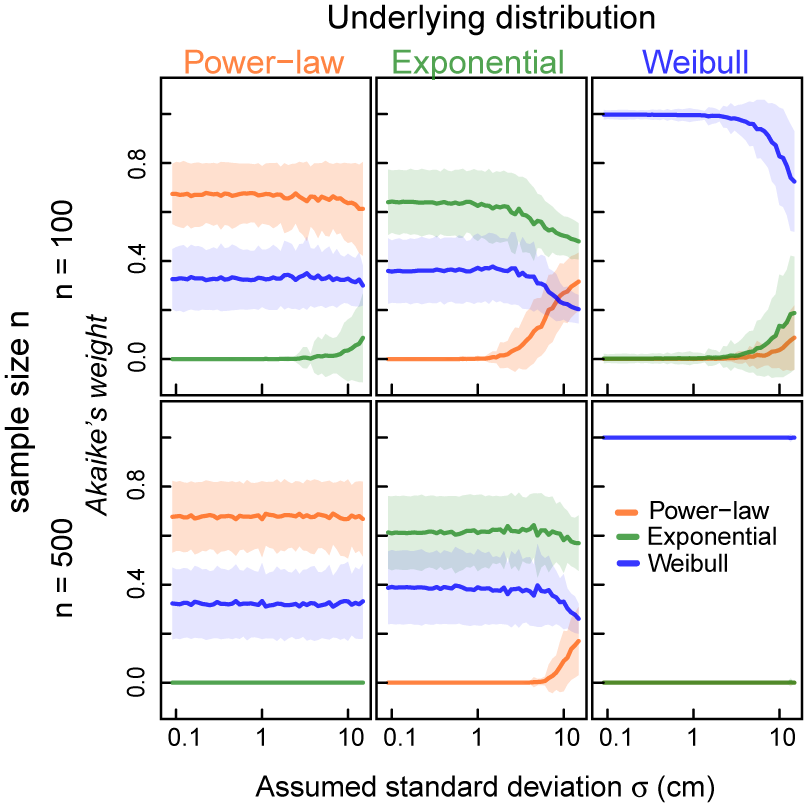

Supplement: Figure S4 — Effect of random measurement errors on Akaike weights with increasing sample size using Gaussian MLE . Weights are calculated with MLE assuming measurement errors (Gaussian MLE) dependent on the Gaussian distributed errors with mean cm and assumed standard deviation (x-axis in cm). The highest Akaike weight determines the best fit of a frequency distribution to the data. The evaluated virtual data sets originate from the three truncated distributions (per column from left to right: power-law, negative exponential and Weibull distribution) which underlie them. Top: Effect of measurement errors on the identification of the correct distribution based on virtual data of sample size n = 100. Bottom: Effect of measurement errors on the identification of the correct distribution based on virtual data of sample size n = 500. Solid lines represent the mean Akaike weights and shaded areas show the standard deviation (of 250 calculated values). (TIF) [file pone.0058036.s004.tif]

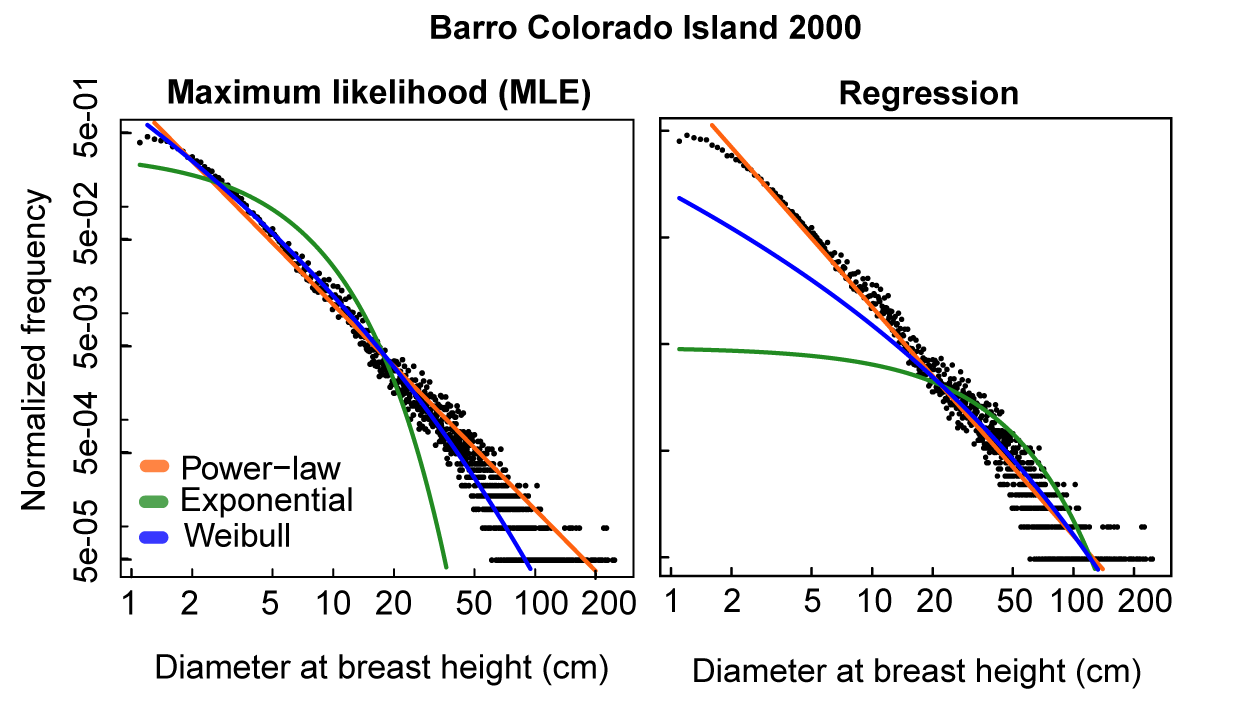

Supplement: Figure S5 — Log-log plots of the fits using regression (right) and Gaussian MLE (left). Data values (inventory data from Barro Colorado Island) of measured stem diameter (cm) at breast height (1.3 m) are shown as black points and fitted truncated distribution functions are represented by solid lines. The straight line denotes the power-law (orange), the slightly curved line refers to the Weibull distribution (blue) and the stronger curved line depicts the negative exponential distribution function (green). Estimated parameters are for (right) regression (power-law), (exponential distribution), and (Weibull distribution) and for (left) Gaussian MLE (power-law), (exponential distribution), and (Weibull distribution). (TIF) [file pone.0058036.s005.tif]
