# Supplementary material for: On the Challenge of Fitting Tree Size Distributions in Ecology
Source: PLoS One. 2013 Feb 28;8(2):e58036. doi: 10.1371/journal.pone.0058036 (PMC3585190; doi:10.1371/journal.pone.0058036)
Supplement: Table S1 — Specific key points of the evaluation of the virtual data samples. (i) bin width b or standard deviation at which the mean bias is greater than or equal to 5% of the true parameter value, (ii) maximum absolute value of the mean bias as percentage of the true parameter value and (iii) the bin width or standard deviation at which the next best distribution reveals the same or a higher mean Akaike weight. We consider the three truncated frequency distributions (power-law, negative exponential and Weibull distribution) and evaluate virtual data samples produced from these distributions using standard MLE (assuming no observation uncertainties), multinomial MLE (correcting binning of data) and Gaussian MLE (correcting measurement errors). (DOC) [file pone.0058036.s006.doc]

**Table S1. Specific key points of the evaluation of the virtual data samples.**

| distribution | Specific key | Binning |  | Measurement | error |
| --- | --- | --- | --- | --- | --- |
| $f\left(x; \theta\right)$ | points | *Standard* | *Multinomial* | *Standard* | *Gaussian* |
|  |  | *MLE* | *MLE* | *MLE* | *MLE* |
| Power-law | Mean bias greater | $b$ > 1.5 | none | $\sigma$ > 0.33 | none |
|  | than or equal to |  |  |  |  |
|  | 5 % of true |  |  |  |  |
|  | parameter for a |  |  |  |  |
|  | bin width $b$ or $\sigma$ |  |  |  |  |
|  | (cm) of: |  |  |  |  |
|  | Max. absolute | 48 % | 0.95 % | 37 % | 0.55 % |
|  | value of mean |  |  |  |  |
|  | bias (% of true |  |  |  |  |
|  | parameter): |  |  |  |  |
|  | Next best | $b$ > 0.67 | none | $\sigma$ > 0.14 | none |
|  | distribution having |  |  |  |  |
|  | the same or a |  |  |  |  |
|  | higher mean |  |  |  |  |
|  | weight for a bin |  |  |  |  |
|  | width $b$ or $\sigma$ (cm) |  |  |  |  |
|  | of: |  |  |  |  |
| Negative | Mean bias greater | $b$ > 1.6 | $b$ > 11 | $\sigma$ > 0.27 | $\sigma$ > 9.9 |
| exponential | than or equal to |  |  |  |  |
|  | 5 % of true |  |  |  |  |
|  | parameter for a |  |  |  |  |
|  | bin width $b$ or $\sigma$ |  |  |  |  |
|  | (cm) of: |  |  |  |  |
|  | Max. absolute | 92 % | 59 % | 84 % | 14 % |
|  | value of mean |  |  |  |  |
|  | bias (% of true |  |  |  |  |
|  | parameter): |  |  |  |  |
|  | Next best | $b$ > 0.91 | $b$ > 27 | $\sigma$ > 0.18 | none |
|  | distribution having |  |  |  |  |
|  | the same or a |  |  |  |  |
|  | higher mean |  |  |  |  |
|  | weight for a bin |  |  |  |  |
|  | width $b$ or $\sigma$ (cm) |  |  |  |  |
|  | of: |  |  |  |  |
| Weibull | Mean bias greater | $b$ > 1 | none | $\sigma$ > 0.08 | none |
|  | than or equal to 5 |  |  |  |  |
|  | % of $\beta$-parameter |  |  |  |  |
|  | for a bin width $b$ |  |  |  |  |
|  | or $\sigma$ (cm) of: |  |  |  |  |
|  | Mean bias greater | $b$ > 1.2 | $b$ > 44 | $\sigma$ > 0.17 | none |
|  | than or equal to 5 |  |  |  |  |
|  | % of $\gamma$-parameter |  |  |  |  |
|  | for a bin width $b$ |  |  |  |  |
|  | or $\sigma$ (cm) of: |  |  |  |  |
|  | Max. absolute | 100 % | 4.8 % | 92 % | 2.8 % |
|  | value of mean |  |  |  |  |
|  | bias (% of $\beta$- |  |  |  |  |
|  | parameter): |  |  |  |  |
|  | Max. absolute | 280 % | 8.6 % | 110 % | 2.4 % |
|  | value of mean |  |  |  |  |
|  | bias (% of $\gamma$- |  |  |  |  |
|  | parameter): |  |  |  |  |
|  | Next best | none | none | $\sigma$ > 12.0 | none |
|  | distribution having |  |  |  |  |
|  | the same or a |  |  |  |  |
|  | higher mean |  |  |  |  |
|  | weight for a bin |  |  |  |  |
|  | width $b$ or $\sigma$ (cm) |  |  |  |  |
|  | of: |  |  |  |  |
