# Supplementary material for: On the Challenge of Fitting Tree Size Distributions in Ecology
Source: PLoS One. 2013 Feb 28;8(2):e58036. doi: 10.1371/journal.pone.0058036 (PMC3585190; doi:10.1371/journal.pone.0058036)
Supplement: Methods S1 — Details on the evaluation procedure and formulas. (DOC) [file pone.0058036.s007.doc]

**Methods S1: Details on the evaluation procedure and formulas**

***Standard MLE***

As perfect observation values of the sample $x=\left(x_{1},...,x_{n}\right)$ are assumed, we maximize the likelihood (eqn 1). Thereby, for MLE that do not consider observation uncertainties the probability $p\left(x; \theta\right)$ is merely replaced by the corresponding density function $f\left(x; \theta\right)$ for each frequency distribution (Table 1):

$$\ln\left(L(x;\theta)\right)=\sum_{i=1}^{n} \ln\left((\alpha-1)\cdot[x_{\min}^{-(\alpha-1)}-x_{\max}^{-(\alpha-1)}]^{-1}\cdot x_{i}^{-\alpha}\right)$$

$$\ln\left(L(x;\theta)\right)=\sum_{i=1}^{n} \ln\left(\lambda\cdot[\exp(-\lambda\cdot x_{\min})-\exp(-\lambda\cdot x_{\max})]^{-1}\cdot\exp(-\lambda\cdot x_{i})\right)$$

$$\ln\left(L(x;\theta)\right)=\sum_{i=1}^{n} \ln\left(\beta\cdot\gamma\cdot[\exp(-\beta\cdot x_{\min}^{\gamma})-\exp(-\beta\cdot x_{\max}^{\gamma})]^{-1}\cdot\exp(-\beta\cdot x_{i}^{\gamma})\cdot x_{i}^{(\gamma-1)}\right)$$

Transforming these likelihoods leads to:

$$\ln\left(L(x;\theta)\right)=n\cdot\ln\left(\alpha-1\right)-n\cdot\ln\left(x_{\min}^{-(\alpha-1)}-x_{\max}^{-(\alpha-1)}\right)-\alpha\cdot\sum_{i=1}^{n} \ln\left(x_{i}\right)$$

$$\ln\left(L(x;\lambda\right)=n\cdot\ln\left(\lambda\right)-n\cdot\ln\left(\exp\left(-\lambda\cdot x_{\min}\right)-\exp\left(-\lambda\cdot x_{\max}\right)\right)-\lambda\cdot\sum_{i=1}^{n} x_{i}$$

$$\ln\left(L\left(x;\beta,\gamma\right)\right)=n\cdot\ln\left(\beta\cdot\gamma\right)-n\cdot\ln\left(\exp\left(-\beta\cdot x_{\min}^{\gamma}\right)-\exp\left(-\beta\cdot x_{\max}^{\gamma}\right)\right)+\left(\gamma-1\right)\cdot\sum_{i=1}^{n} \ln\left(x_{i}\right)-\beta\cdot\sum_{i=1}^{n} x_{i}^{\gamma}$$

***Multinomial MLE***

Following (eqn 1) we also maximize the likelihood, but the probability $p\left(x; \theta\right)$ accounting for binning of data is now expressed by the multinomial distribution including the corresponding density function $f\left(x; \theta\right)$ of the assumed frequency distributions. Thereby $n$ denotes the sample size, $N_{j}$ the number of observations falling in the bin $j$ with $j=1,...,q$ and $q$ the total number of bins. Bins are half-open intervals $\left[B_{j};B_{j}+b\right)$ of width $b$ (cm) and start at $B_{1}=x_{\min}$ (cm). For each bin a theoretical probability, describing an observation value to fall within that bin, is calculated based on the assumed distribution with its density function $f\left(x; \theta\right)$:

$$\tilde{f}(x;\alpha):=\int_{B_{j}}^{B_{j}+b} f(x;\alpha)dx=\frac{B_{j}^{-(\alpha-1)}-\left(B_{j}+b\right)^{-(\alpha-1)}}{x_{\min}^{-(\alpha-1)}-x_{\max}^{-(\alpha-1)}}$$

$$\tilde{f}(x;\lambda):=\int_{B_{j}}^{B_{j}+b} f(x;\lambda)dx=\frac{\exp\left(-\lambda\cdot B_{j}\right)-\exp\left(-\lambda\cdot\left(B_{j}+b\right)\right)}{\exp\left(-\lambda\cdot x_{\min}\right)-\exp\left(-\lambda\cdot x_{\max}\right)}$$

$$\tilde{f}(x;\beta,\gamma):=\int_{B_{j}}^{B_{j}+b} f(x;\beta,\gamma)dx=\frac{\exp\left(-\beta\cdot B_{j}^{\gamma}\right)-\exp\left(-\beta\cdot\left(B_{j}+b\right)^{\gamma}\right)}{\exp\left(-\beta\cdot x_{\min}^{\gamma}\right)-\exp\left(-\beta\cdot x_{\max}^{\gamma}\right)}$$

For each density function of the three distributions we then get the following specific *likelihood*:

***Gaussian MLE***

Following (eqn 1) we maximize the likelihood according to the probability $p\left(x; \theta\right)$, which is expressed by the convolution of an assumed truncated Gaussian distribution for measurement errors $\varepsilon_{meas}\sim N\left(\mu=0; \sigma>0\right)$ and the corresponding density function $f\left(x; \theta\right)$ of the assumed frequency distribution (Table 1):

$\ln\left(L\left(x;\theta\right)\right)=\sum_{i=1}^{n} \ln\left(\int_{\max\left(x_{\min};x_{i}-3\cdot\sigma\right)}^{\min\left(x_{\max};x_{i}+3\cdot\sigma\right)} \frac{f\left(x;\theta\right)}{\sigma\cdot erf\left(3/\sqrt{2}\right)\cdot\sqrt{2\cdot\pi}}\cdot\exp\left(-\frac{\left(x_{i}-x\right)^{2}}{2\cdot\sigma^{2}}\right)dx\right)$,

where $erf()$ represents the Gauss error function.

**Random number generators for the considered frequency distributions**

Using the *inverse transformation method* to generate virtual data values from an assumed frequency distribution $f\left(x; \theta\right)$, the inverse of the cumulative distribution function $F^{-1}\left(r; \theta\right)$, also known as the $r$-quantile, is calculated. On the basis of a randomly produced number $r\in\left[0; 1\right]$ drawn from a uniform distribution, we calculated the inverse of the cumulative distribution function in the following manner, so that it is easier to solve them afterwards according to $x$:

$$1-F(x;\theta)=1-r \to\left[1-F\right]^{-1}\left(1-r;\theta\right)=x$$

This results in the following random number generator (or $r$-quantiles) for each distribution:

$$x_{Power-law}=\left[\left(1-r\right)\cdot\left(x_{\min}^{-(\alpha-1)}-x_{\max}^{-(\alpha-1)}\right)+x_{\max}^{-(\alpha-1)}\right]^{-1/(\alpha-1)}$$

$$x_{Exponential}=-\frac{1}{\lambda}\cdot\ln\left(\left(1-r\right)\cdot\left(\exp\left(-\lambda\cdot x_{\min}\right)-\exp\left(-\lambda\cdot x_{\max}\right)\right)+\exp\left(-\lambda\cdot x_{\max}\right)\right)$$

$$x_{Weibull}=\left[-\frac{1}{\beta}\cdot\ln\left(\left(1-r\right)\cdot\left(\exp\left(-\beta\cdot x_{\min}^{\gamma}\right)-\exp\left(-\beta\cdot x_{\max}^{\gamma}\right)\right)+\exp\left(-\beta\cdot x_{\max}^{\gamma}\right)\right)\right]^{1/\gamma}$$
