# Supplementary material for: On the Challenge of Fitting Tree Size Distributions in Ecology
Source: PLoS One. 2013 Feb 28;8(2):e58036. doi: 10.1371/journal.pone.0058036 (PMC3585190; doi:10.1371/journal.pone.0058036)
Supplement: Code S1 — R-script of MLE evaluation on the example of Barro Colorado Island census year 2000. (DOC) [file pone.0058036.s008.doc]

**#Code S1**

##################################################################### R-code of MLE evaluation

# on the example of Barro Colorado Island census year 2000

#

# written in R 2.10.0

# authors: Franziska Taubert, Florian Hartig,

# Hans-Jürgen Dobner, Andreas Huth

#

# date: 18. June 2012

#

####################################################################

#--------------------------------------------------

#functions needed for the evaluations

erf <- function(x) 2*pnorm(x*sqrt(2))-1

weight <- function(aics){

AICmin <- min(aics[1],aics[2],aics[3])

wP <- exp(-(aics[1] - AICmin)/2.0)

wE <- exp(-(aics[2] - AICmin)/2.0)

wW <- exp(-(aics[3] - AICmin)/2.0)

wsum <- (wP + wE + wW)

return (c(wP/wsum, wE/wsum, wW/wsum))

}

#--------------------------------------------------

# Read in data file

# Census data of Barro Colorado Island can be requested at CTFS

# (see Article for reference)

# Please ensure that data file is in the correct format (*.txt)

# use setwd() to set the directory where the data file is located, #if needed, e.g. setwd("C:/")

bci2000 <- read.delim("PlotsData2000.txt",sep="\t",dec=".")

####################################################################

# Select and transform measurement data:

# a) Delete measurements of trees unfavourable for evaluation

# here: dead trees, secondary stems and diameter measurements

# marked as NA

bci2000_1 <- subset(bci2000,bci2000[,16]=="alive")

bci2000_2 <- subset(bci2000_1,bci2000_1[,15]=="main")

bci2000_3 <- subset(bci2000_2,is.na(bci2000_2[,11])==F)

# b) Change unit of stem diameters from (mm) to (cm)

bci2000_4 <- bci2000_3[,11]/10.

# c) remove all diameter smaller than 1.05 cm

bci2000_d <- subset(bci2000_4, bci2000_4 >= 1.05) # represent now xi: observation sample in (cm)

# c) Estimate xmin and xmax by the minimum and maximum

# measured data values

xmin = min(bci2000_d)

xmax = max(bci2000_d)

sample_size <- length(bci2000_d) # represents now n: sample size of data set = total number of

# observations

####################################################################

# Evaluation

# -------------------------------------

# 1) Fit using standard MLE

# (not considering observation uncertainties)

#--------------------------------------

### a) assuming a power-law distribution for describing

# the stem size distribution

# implementation of the likelihood of standard MLE

# for a power-law assumption

standard_likelihood_P <- function(alpha){

param <- -(alpha - 1)

return (sum(log((-param/((xmin^param)-

(xmax^param)))*(bci2000_d^(param-1)))))

}

solver <- optimize(standard_likelihood_P,c(1.001,20),maximum=T) # maximizing the likelihood for a power-law assumption # maximum is searched within the parameter range [1.001; 20.0]

# for the parameter alpha # if convergence problems occur, please try to change the limits of

# this parameter range

estimate_alpha <- solver[[1]] # parameter estimate for alpha

AIC_Powerlaw <- (-2.0*standard_likelihood_P(estimate_alpha)) + 2.0 # Akaike Information Criterium for the Power-law assumption

### b) assuming a negative exponential distribution for describing

# the stem size distribution

# implementation of the likelihood of standard MLE for a negative

# exponential distribution assumption

standard_likelihood_E <- function(lambda){

param <- -lambda

return (sum(log((-param/(exp(param*xmin)-

exp(param*xmax)))*exp(param*bci2000_d))))

}

solver <- optimize(standard_likelihood_E,c(0.0001,1.0),maximum=T); # maximizing the likelihood for a negative exponential distribution

# assumption # maximum is searched within the parameter range [0.0001; 1.0] for

# lambda # if convergence problems occur, please try to change the limits of

# this parameter range

estimate_lambda <- solver[[1]] # parameter estimate for lambda

AIC_Exponential <- (-2.0*standard_likelihood_E(estimate_lambda))+2.0

# Akaike Information Criterium for the negative exponential

# distribution assumption

### c) assuming a Weibull distribution for describing the stem size

# distribution

# implementation of the likelihood of standard MLE for a Weibull

# distribution assumption

standard_likelihood_W <- function(param){

beta_p <- param[1]

gamma_p <- param[2]

(-1)*sum(log(((beta_p*gamma_p)/(exp(-beta_p*(xmin^gamma_p))

-exp(-beta_p*(xmax^gamma_p))))*(bci2000_d^(gamma_p - 1))

*exp(-beta_p*(bci2000_d^gamma_p))))

}

solver <- optim(c(0.5,0.5),standard_likelihood_W) # maximizing the likelihood for a Weibull distribution assumption # maximum is searched by using the starting values 0.5 for each

# parameter (beta, gamma) # if convergence problems occur, please try to change starting

# values # or see help for further options for 'optim' (e.g. change

# optimization algorithm etc.)

estimate_beta <- solver[[1]][1] # parameter estimate for beta

estimate_gamma <- solver[[1]][2] # parameter estimate for gamma

AIC_Weibull <- (2.0*standard_likelihood_W(solver[[1]])) + 4.0; # Akaike Information Criterium for the Weibull distribution

# assumption

### summarizing all parameter estimates in a vector

standardL_estimates <- c(estimate_alpha,estimate_lambda,estimate_beta,estimate_gamma)

### summarizing the 3 Akaike's weights of the distribution

# assumptions in a vector

standardL_weights <- weight(c(AIC_Powerlaw,AIC_Exponential,AIC_Weibull))

# -------------------------------------

# 2) Fit using multinomial MLE (considering pre-binning of data)

#--------------------------------------

bin_width = 0.1 # represents now b: bin width in (cm)

# counting the number of observations for each distinct measured

# stem diameter

breaks_defined <- seq(xmin - (bin_width/2.0), xmax + (bin_width/2.0), bin_width);

# breaks of the bins [) for setting up the histogram are defined

hist_counts <- hist(bci2000_d, breaks = breaks_defined, plot = F, right=F, include.lowest=F)

# the histogram is produced delivering the absolute counts of

# observations per bin

data_bins <- array(rbind(hist_counts[[2]], hist_counts[[1]][1:(length(hist_counts[[1]])-1)], hist_counts[[1]][2:length(hist_counts[[1]])]) ,c(3,length(hist_counts[[2]])))

# data matrix is produced as input for the likelihood functions

# 1st column: counts

# 2nd column: left limit of bin [Bj; Bj + b)

# 3rd column: right limit of bin [Bj; Bj + b) # rows correspond to the bins

### a) assuming a power-law distribution for describing the stem

# size distribution

# implementation of the likelihood of multinomial MLE for a power-

# law distribution assumption

multinomial_likelihood_P <- function(alpha){

param <- -(alpha - 1)

return (sum(data_bins[1, ]*

log(((data_bins[2,]^param)-(data_bins[3, ]^param))/

((xmin^param)-(xmax^param)))))

}

solver <- optimize(multinomial_likelihood_P,c(1.001,5),maximum=T) # maximizing the likelihood for a power-law distribution assumption # maximum is searched by in the parameter range [1.001; 20.0] for

# alpha # if convergence problems occur, please try to change the limits of

# this range

estimate_alpha <- solver[[1]] # parameter estimate for alpha

AIC_Powerlaw <- (-2.0*dmultinom(hist_counts[[2]],sample_size,

((data_bins[2,]^(-(estimate_alpha - 1.)))

-(data_bins[3,]^(-(estimate_alpha - 1.))))

/((xmin^(-(estimate_alpha - 1.)))

-(xmax^(-(estimate_alpha - 1.)))), log=T)) + 2.0

# Akaike Information Criterium for the power-law distribution

# assumption

### b) assuming a negative exponential distribution for describing

# the stem size distribution

# implementation of the likelihood of multinomial MLE for a

# exponential distribution assumption

multinomial_likelihood_E <- function(lambda){ param <- -lambda

return (sum(data_bins[1,]*

log((exp(param*data_bins[2,])-

exp(param*data_bins[3,]))/

(exp(param*xmin)-exp(param*xmax)))))

}

solver <- optimize(multinomial_likelihood_E,c(0.,2.0),maximum=T) # maximizing the likelihood for a negative exponential distribution

# assumption # maximum is searched by in the parameter range [0.0; 2.0] for

# lambda # if convergence problems occur, please try to change the limits of

# this range

estimate_lambda <- solver[[1]] # parameter estimate for lambda

AIC_Exponential <- (-2.0*dmultinom(hist_counts[[2]],sample_size,

(exp(-estimate_lambda*data_bins[2,])

-exp(-estimate_lambda*data_bins[3,]))

/(exp(-estimate_lambda*xmin)

-exp(-estimate_lambda*xmax)),log=T)) + 2.0

# Akaike Information Criterium for the negative exponential

# distribution assumption

### c) assuming a Weibull distribution for describing the stem size

# distribution

# implementation of the likelihood of multinomial MLE for a Weibull

# distribution assumption

multinomial_likelihood_W <- function(param){

beta_p <- -param[1]

gamma_p <- param[2]

return ((-1)*sum(data_bins[1, ]*

log((exp(beta_p*(data_bins[2,]^gamma_p))

-exp(beta_p*(data_bins[3,]^gamma_p)))

/(exp(beta_p*(xmin^gamma_p))

-exp(beta_p*(xmax^gamma_p))))))

}

solver <- optim(c(0.5,0.5),multinomial_likelihood_W) # maximizing the likelihood for a Weibull distribution assumption # maximum is searched by using the starting values 0.5 for each

# parameter (beta, gamma) # if convergence problems occur, please try to change starting

# values # or see help for further options for 'optim' (e.g. change

# optimization algorithm etc.)

estimate_beta <- solver[[1]][1] # parameter estimate for beta

estimate_gamma <- solver[[1]][2] # parameter estimate for gamma

AIC_Weibull <- (-2.0*dmultinom(hist_counts[[2]],sample_size,

(exp(-estimate_beta*(data_bins[2,]^estimate_gamma))

-exp(-estimate_beta*(data_bins[3,]^estimate_gamma)))

/(exp(-estimate_beta*(xmin^estimate_gamma))

-exp(-estimate_beta*(xmax^estimate_gamma))),log=T)) +

4.0

# Akaike Information Criterium for the Weibull distribution

# assumption

### summarizing all parameter estimates in a vector

multinomialL_estimates <- c(estimate_alpha,estimate_lambda,estimate_beta,estimate_gamma)

### summarizing the 3 Akaike's weights of the distribution

# assumptions in a vector

multinomialL_weights <- weight(c(AIC_Powerlaw,AIC_Exponential,AIC_Weibull))

# -------------------------------------

# 3) Fit using Gaussian MLE (considering measurement errors)

#

# likelihood function changes slightly according to the estimated

# measurement error of Chave et al. (2004)

#

# change from: integral(f(x;theta)*N(0;sigma))

# to: integral(f(x;q)*[0.95*N(0;0.0062*diameter + 0.0904) +

# 0.05*N(0;4.64)])

#

# where N(mu,sigma) denotes the Gaussian distribution with

# mean=mu and standard deviation=sigma

#--------------------------------------

data_array <- array(bci2000_d,c(1,length(bci2000_d)))

### a) assuming a power-law distribution for describing the stem

# size distribution

convolution_P <- function(daten, param, sigma_index){

if(sigma_index == 1) sigma <- (0.0062*daten) + 0.0904

if(sigma_index == 2) sigma <- 4.64

integral <- integrate(function(x) ((param-1)*

(((xmin^(-(param-1)))-(xmax^(-(param-1))))^(-

1))*(x^(-param))*(1/(sigma*sqrt(2*pi)*

erf(3/sqrt(2))))*exp(-((daten-

x)^2)/(2*(sigma^(-2)))))

,lower=max(xmin,daten-(3*sigma)),

upper=min(xmax,daten+(3*sigma)))[[1]]

return (integral)

}

# implementation of the likelihood of Gaussian MLE for a power-law

# distribution assumption

gaussian_likelihood_P <- function(alpha){

return (sum(log((0.95*apply(data_array,2,convolution_P,param

= alpha,sigma_index = 1))

+ (0.05*apply(data_array,2,convolution_P,param =

alpha,sigma_index = 2)))))

}

solver <- optimize(gaussian_likelihood_P,c(1.01,8),maximum=T) # maximizing the likelihood for a power-law distribution assumption # maximum is searched in the parameter range [1.01, 8] for alpha # if convergence problems occur, please try to change this range

estimate_alpha <- solver[[1]]; # parameter estimate for alpha

AIC_Powerlaw <- (-2.0*gaussian_likelihood_P(estimate_alpha)) + 2.0 # Akaike Information Criterium for the power-law distribution

# assumption

### b) assuming a negative exponential distribution for describing

# the stem size distribution

convolution_E <- function(daten, param, sigma_index){

if(sigma_index == 1) sigma <- (0.0062*daten) + 0.0904

if(sigma_index == 2) sigma <- 4.64

integral <- integrate(function(x) (param*

((exp(-param*xmin)-exp(-param*xmax))^(-

1))*exp(-param*x)*(1/(sigma*sqrt(2*pi)*

erf(3/sqrt(2))))*exp(-((daten-

x)^2)/(2*(sigma^(-2)))))

,lower=max(xmin,daten-(3*sigma)),

upper=min(xmax,daten+(3*sigma)))[[1]]

return (integral);

}

# implementation of the likelihood of Gaussian MLE for an

# exponential distribution assumption

gaussian_likelihood_E <- function(lambda){

return (sum(log((0.95*apply(data_array,2,

convolution_E,param = lambda,sigma_index = 1))

+ (0.05*apply(data_array,2,convolution_E,param =

lambda,sigma_index = 2)))))

}

solver <- optimize(gaussian_likelihood_E,c(0.0,2.0),maximum=T) # maximizing the likelihood for an exponential distribution

# assumption # maximum is searched in the parameter range [0.0, 2.0] for lambda # if convergence problems occur, please try to change this range

estimate_lambda <- solver[[1]]; # parameter estimate for lambda

AIC_Exponential <- (-2.0*(gaussian_likelihood_E(estimate_lambda))) + 2.0

# Akaike Information Criterium for the exponential distribution

# assumption

### c) assuming a Weibull distribution for describing the stem size

# distribution

convolution_W <- function(daten, param1, param2, sigma_index){

if(sigma_index == 1) sigma <- (0.0062*daten) + 0.0904

if(sigma_index == 2) sigma <- 4.64

integral <- integrate(function(x) (param1*param2*

((exp(-param1*(xmin^param2))-exp(-

param1*(xmax^param2)))^(-1))*(x^(param2-

1))*exp(-param1*(x^param2))*

(1/(sigma*sqrt(2*pi)*erf(3/sqrt(2))))*exp(-

((daten-x)^2)/(2*(sigma^(-2)))))

,lower=max(xmin,daten-(3*sigma)),

upper=min(xmax,daten+(3*sigma)))[[1]]

return (integral);

}

# implementation of the likelihood of Gaussian MLE for a Weibull

# distribution assumption

gaussian_likelihood_W <- function(param){

beta_p <- param[1]

gamma_p <- param[2]

(-1)*sum(log((0.95*apply(data_array,2,convolution_W,param1

= beta_p,param2 = gamma_p,sigma_index = 1))

+ (0.05*apply(data_array,2,convolution_W,param1 =

beta_p,param2 = gamma_p,sigma_index = 2))))

}

solver <- optim(c(1.5,0.5),gaussian_likelihood_W) # maximizing the likelihood for a Weibull distribution assumption # maximum is searched by using the starting values 0.5 for each

# parameter (beta, gamma) # if convergence problems occur, please try to change starting

# values # or see help for further options for 'optim' (e.g. change

# optimization algorithm etc.) # e.g. if (default setted) Nelder-Mead-Algorithm does not converge,

# one could try the L-BFGS-B-Algorithm, e.g.: # solver <- optim(c(0.5,0.5),gaussian_likelihood_W,lower=c(10^(-

# 8),10^(-8)),method="L-BFGS-B")

estimate_beta <- solver[[1]][1] # parameter estimate for beta

estimate_gamma <- solver[[1]][2] # parameter estimate for gamma

AIC_Weibull <- (2.0*(gaussian_likelihood_W(solver[[1]]))) + 4.0 # Akaike Information Criterium for the Weibull distribution

# assumption

### summarizing all parameter estimates in a vector

gaussianL_estimates <- c(estimate_alpha,estimate_lambda,estimate_beta,estimate_gamma)

### summarizing the 3 Akaike's weights of the distribution

# assumptions in a vector

gaussianL_weights <- weight(c(AIC_Powerlaw,AIC_Exponential,AIC_Weibull))

####################################################################

# Plotting measurement data with results here e.g. those of MLE

# using the multinomial MLE (correcting binning)

midpoints <- seq(xmin -(bin_width/2.0),xmax + (bin_width/2.0),bin_width)

# plotting each observation value

counter <- hist(bci2000_d,plot=F,breaks=midpoints,include.lowest=T)

nonzero <- which(counter$count > 0)

# plot of observed data points

plot(x = counter$mids[nonzero],y = (counter$count[nonzero])/(sample_size*1),

log="xy",xlab="Diameter at breast height (cm)", ylab="Normalized relative frequency", type="p", pch=20,

main="BCI 2000")

# plot of power-law fit

yvec2 <- (multinomialL_estimates[1] - 1)*(((xmin^(-(multinomialL_estimates[1] - 1)))-(xmax^(-(multinomialL_estimates[1] - 1))))^(-1))*((counter$mids[nonzero])^(-multinomialL_estimates[1])) lines(x=counter$mids[nonzero],y=yvec2,col=rgb(255,98,15,255,maxColorValue=255),lwd=2,lty=1)

# plot of fit of negative exponential distribution

yvec3 <- multinomialL_estimates[2]*((exp(-multinomialL_estimates[2]*xmin)-exp(-multinomialL_estimates[2]*xmax))^(-1))*exp(-multinomialL_estimates[2]*counter$mids[nonzero])

lines(x=counter$mids[nonzero],y=yvec3,col=rgb(34,139,34,255,maxColorValue=255),lwd=2,lty=1)

# plot of Weibull fit

yvec4 <- (multinomialL_estimates[3]*multinomialL_estimates[4])*((exp(-multinomialL_estimates[3]*(xmin^multinomialL_estimates[4]))

-exp(-multinomialL_estimates[3]*(xmax^multinomialL_estimates[4])))^(-1))*exp(-multinomialL_estimates[3]*((counter$mids[nonzero])^multinomialL_estimates[4]))*((counter$mids[nonzero])^(multinomialL_estimates[4]-1)) lines(x=counter$mids[nonzero],y=yvec4,col=rgb(0,0,255,255,maxColorValue=255),lwd=2,lty=1)

#the position of the legend may have to be adjusted according to #your data

xpos = 1

ypos = 0.5

legend(xpos, ypos, c("Power-law","Neg. exponential","Weibull"),

col = c(rgb(255,98,15,255,maxColorValue=255),rgb(34,139,34,255,maxColorValue=255),rgb(0,0,255,255,maxColorValue=255)))
